# A Numerical Model for Predicting the Effect of Tool Nose Radius on Machining Process Performance during Orthogonal Cutting of AISI 1045 Steel

**DOI:** 10.3390/ma15093369

**Published:** 2022-05-08

**Authors:** Zakaria Ahmed M. Tagiuri, Thien-My Dao, Agnes Marie Samuel, Victor Songmene

**Affiliations:** Department of Mechanical Engineering, École de Technologie Supérieure (ÉTS) 1100 Notre-Dame Street West, Montréal, QC H3C 1K3, Canada; zakaria-ahmed-m.tagiuri.1@ens.etsmtl.ca (Z.A.M.T.); thien-my.dao@etsmtl.ca (T.-M.D.); agnes-marie.samuel@etsmtl.ca (A.M.S.)

**Keywords:** tool nose radius, orthogonal cutting, AISI 1045, cutting forces, temperature, stress, tool wear

## Abstract

This paper presents the development of a numerical model for predicting and studying the effects of tool nose geometries and its interactions with cutting parameters during orthogonal cutting of AISI 1045 steel. The process performance characteristics studied were cutting temperature, effective stress, cutting forces and tool wear. The cutting simulations were done using the commercial DEFORM-2D ^R^ V 11.3 software, based on the finite element method (FEM). The cutting tool used had a round nose with various nose radii (0.01–0.9 mm), while the machining parameters tested were the feed rate (0.1–0.3 mm/rev), the cutting speed (100–500 m/min) and the rake angle (–5° to +10°). The interactions between the tool nose radius and the cutting parameters (speed, feed) were found to affect mostly the cutting stress and, slightly, the tool wear rate. These interactions did not much influence the cutting temperature, that was found to be high when the tool nose radius and/or the cutting speed were high. The maximum temperature was found to occur at the middle of the tool-chip contact length and at the interaction of nose radius and flank face of the tool. Except for some fluctuations, there was no significant difference in tool wear rate between small and large nose radius scales.

## 1. Introduction

One of the major problems commonly met on a continual basis during cutting processes in different machining industries is failure of the cutting tool, due to excessive mechanical forces, higher temperatures and gradual tool wear. Gradual tool wear occurs on the cutting tool edge, especially for complex parts that may be difficult to cut, leading to short tool life, and, consequently, increasing cycle time and machining costs. To enable machining companies to become more competitive, there is an imminent need to incorporate cutting-edge preparation as a new concept in the context of cutting tools, by generating a specific geometry around the cutting tool edge, so as to improve tool life, part quality, and reduce cycle time and machining cost [1,2]. In literature, few research studies have been conducted on machining operations by adopting cutting edge preparation approaches that show their advantages in different machining applications. Denkena and Biermann [3] reported that a good selection of cutting tool edge geometry influences machining process performance. It improves tool wear resistance, and tool life, as well as having effects on chip formation, and mechanical and thermal stresses. Biermann et al. [4] proposed a finite element analysis-based methodology of the optimization of prepared cutting-edge micro forms that reduce tool wear during the machining of Inconel 718. They found that an asymmetrical micro shape is the optimal cutting-edge profile, by investigating cutting-edge parameters S_α_, S_β_ and S_γ_. In fact, M. Shnfir et al. [5] investigated the machinability of hardened steel AISI 1045 during face milling using cutting tools with tool edge preparation, in order to identify the effects of cutting parameters, milling configuration, edge preparation and work material hardness on cutting force, power consumption and flank tool wear. Their results showed that lower feed rates and increased workpiece hardness resulted in decreasing cutting forces with honed edge inserts. At low cutting speeds and high feed rates, flank wear was increased, and the use of tools with chamfered tool-edge preparation greatly improved tool wear. Z. Weiwei et al. [6] analysed the effects of cutting-edge micro-geometry on surface roughness and white layer formation in turning AISI 52100 steel. They found that increasing chamfer width improved surface roughness, but increasing the chamfer angle did not have any significant effect. Moreover, large rounded and chamfered edges could contribute to white layer formation.

Despite the increase in application of cutting tools with round geometry preparation techniques in current developments, previous research investigations on cutting edge geometry preparation, and its effect on the performance of machining processes, are very limited. M. Javidikia et al. [7] studied interactive impacts between cutting-edge radius and cutting speed, feed rate, and rake angle on machining forces, cutting temperature, and chip thickness during turning of aluminium alloy 6061-T6. In fact, by comparing conventional machining (CM) with high-speed machining (HSM), the obtained results showed that both cutting force and feed force are increased when the cutting-edge radius is increased at low cutting speeds (related to conventional machining (CM)) and at a negative rake angle. For the thermal aspect, the maximum cutting temperatures remain almost constant with increasing cutting speed but they increase with increasing edge radius for all the cutting speeds considered, whereas an increase in the average temperatures of the tool tip is observed in the case of high-speed machining (HSM). Moreover, the maximum cutting temperature was found to depend mostly on cutting conditions and tool geometry for the workpiece and tool materials tested. In terms of chip formation, the authors found no significant variation of chip thickness with cutting-edge radius in high-speed machining (HSM) in their study. Zhuang et al. [8] proposed a numerical simulation model to predict the impacts of chamfer angle, chamfer length and feed rate on the machining force of Ti6Al4V. The obtained results showed a significant influence was exhibited by chamfer length and angle on cutting force. However, few studies have been realised on the interaction between cutting speed, edge radius, feed rate and rake angle and their effects on the machining properties of steel, in particular, carbon steel materials. A numerical study was conducted by Cheng et al. [9] on the influence of tool edge radius and rake angle during orthogonal cutting of Fe-Cr-Ni stainless steel on machining performance. They found that stress was more affected than temperature by edge radius, and with increasing rake angle there were fluctuations in terms of stress and a gradual increase in higher temperature. Wang and Jiang [10] performed a numerical research, based on the finite element method, investigating effects of different wiper tool edge designs on temperature and force during orthogonal cutting of AISI 4340 steel. They found that the tool temperature of the flank face is more reduced with wiper tools than with round conventional tools; whereas temperature and force increased on the rake face.

It is stated that on one hand, only the impact of cutting tool radius on different machining output variables was assessed, showing interactive behaviour with various cutting speeds, rake angles, and feed rates. On the other hand, only round edge geometry was considered, and other edge geometries were ignored. Moreover, the study was limited to small tool edge radius. Thus, large tool edge radius has not yet been studied, which points to another lack of data for the macro machining industry.

Based on the above details of the available data, the need to study more interactions between tool geometry and machining parameters for available cutting tool edge preparations is justified. This approach could help tool designers, and guide manufacturing engineers, in selecting a process with optimal geometrical parameters, and establish standard and accurate tool edge preparation procedures applicable in the machining industry.

The objective of the present research is to study the interactive effects of cutting tool geometrical parameters and different machining process conditions for designed tool nose geometry on cutting force, cutting stress, tool wear and temperature distribution. A numerical model, based on finite element analysis, was developed to predict the effects of interactions between cutting nose geometry and machining parameters of feed rate and cutting speed on machining process performance indicators; namely, cutting forces, temperature, tool wear and cutting stress.

## 2. Numerical Simulation Model for Orthogonal Cutting Analysis

**Figure 1:**
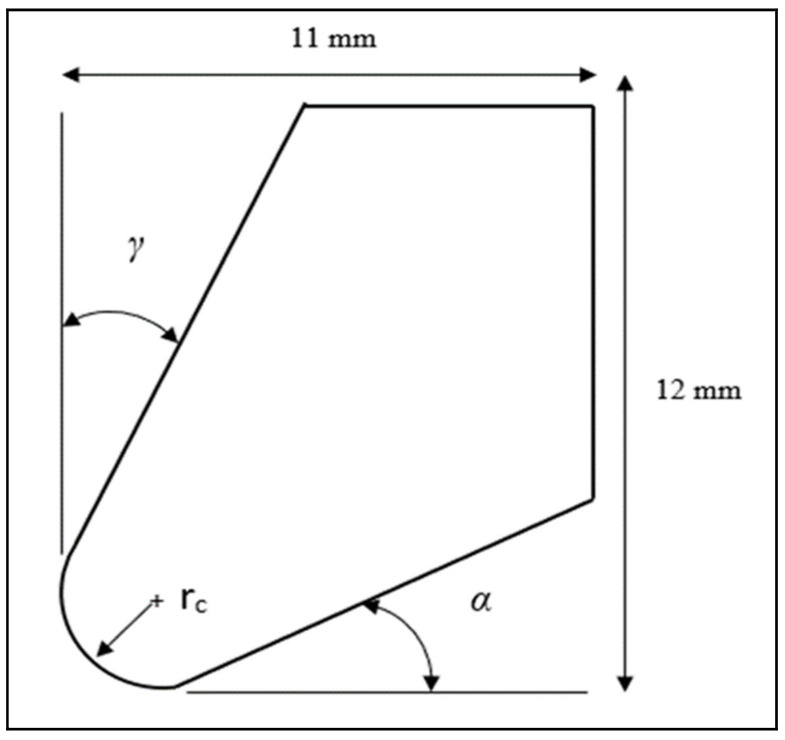
Tool model geometry (*r_c_*, α, *γ* are the nose radius, the clearance angle and the rake angle, respectively).

**Figure 2:**
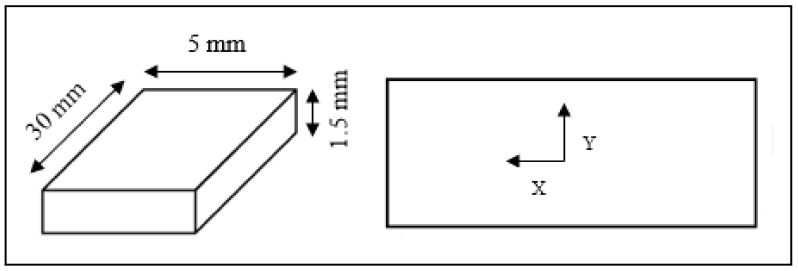
Workpiece model geometry.

The objective of the present simulation study is to predict the effects of cutting tool nose geometries for different edge preparations on machining process performance in terms of temperature distribution, tool wear, effective stresses and strains. Two-dimensional (2D) orthogonal external cutting simulations, with plain strain condition, were conducted using the commercial Finite Element Method (FEM) DEFORM-2D ^R^ V 11.3 software. This software program is based on the Lagrangian formulation for plastic deformation analysis. The material of the workpiece is modelled as a plastic material, whereas the cutting tool is considered as an elastic material. The simulations are limited to round tool geometry. Chip formation is simulated by plastic flow, whereas separation of formed chips from the workpiece is completed by continuous remeshing. It is assumed that there is no crack initiation detected in the tool tip during the cutting process. In the present study, for cutting nose preparation, only round nose geometry was proposed. Figure 1 and Figure 2 show the workpiece and the tool model designs, respectively.

### 2.1. Material Characterization

The selected workpiece material used for the present study is AISI 1045 steel. For the cutting tool, the material used is uncoated cemented carbide. The cutting process occurred in a very short time (around ms), so that the temperature reached the steady state very quickly, thereby causing little variation in thermal conductivity with temperature. So, these properties are supposed constant. Table 1 presents the thermal and physical properties, while Table 2 presents the mechanical properties of the workpiece and tool materials.

**Figure 3:**
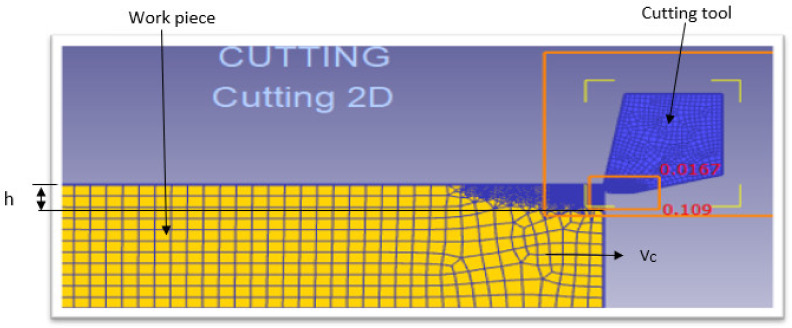
Meshing of the model for cutting simulation at *V*_c_ = 104 m/min, *f* = 0.1 mm/rev.

The numerical model consists of conducting the orthogonal cutting simulations using the commercial DEFORM 2D Finite Element Method software. The cutting geometry model is meshed with two-dimensional (2D) quadrilateral elements. The Finite Element Method (FEM) mesh was based on quadrilateral elements because they can be used with adaptive mesh refinement. This approach generates simple meshes and ensures good control of the density of the elements. Each element has four nodes and can be generated automatically by a default algorithm for solid modeling. Figure 3 shows the meshing of the model for cutting simulation, where *V*_c_, *f* and h are the cutting speed, the feed rate and the depth of cut, respectively.

The transient heat transfer and motion equations used by the DEFORM software during the cutting process may be expressed as [7]:

(1) $$\left[C_{T}]\{\dot{T}\}+[K_{T}]\{T\}=\{Q_{g}\right\}$$

(2) $$\left[M]\{\ddot{U}\}+\{R_{\mathrm{int}}\}=\{R_{\mathrm{ext}}\right\}$$

where [*C_ᴛ_*] is the volume heat capacitance, [*K_ᴛ_*] is the thermal conduction matrices, {Q*_g_*} is the total heat generated, {$\ddot{U}$} is the acceleration vector, {*U*} is the displacement, {*R_int_*} is the vector of internal force, whereas {*R_ext_*} is the vector of external force.

Considering the selected materials, the flow stress model for these materials may be expressed according to the Johnson-Cook constitutive equation as follows [7]:

(3) $$\bar{\sigma }=\left[A+B\left(\varepsilon^{n}\right)\right]\left[1+C\cdot \ln(\frac{\dot{\varepsilon }}{\varepsilon_{0}})\right]\left[1-\frac{\left(T-T_{room}\right)}{{\left(T_{melt}-T_{room}\right)}^{m}}\right]$$

where *ԑ* is the plastic strain $\dot{\varepsilon }$ (s^−1^) is the plastic strain rate, $\varepsilon_{0}$ (^−1^) is the reference plastic strain rate, *T* (°C) is the workpiece temperature, *T_melt_* (°C) is the melt temperature, *T_room_* (°C) is the room temperature, *A* (MPa) is the initial yield strength, and *B* (MPa) is the hardening modulus.

The numerical simulation of tool wear by 2D DEFORM is based on nodal displacement (at each node) caused by wear for a certain cutting time increment. The tool wear is expressed by its proper wear rate at every node, where the wear rate is given by the volume loss of material removed by machining per unit area and per unit time. For this study, only flank wear, which occurred on the flank area, was considered [13].

The tool wear was simulated using the Usui wear model as follows [14]:

(4) $$W=\int A\cdot P\cdot V\cdot e^{-\frac{B}{T}}\cdot dt$$

where *W*, *P*, *V*, *T* and *dt* are tool wear, interface pressure, sliding velocity, temperature and time increment, respectively. *A* and *B* are constants determined experimentally.

#### Boundary Conditions

The displacement and thermal boundary conditions for work piece and tool simulation are given as follows:

The tool is fixed, and the work piece is being cut at the specified cutting speed:

- For heat transfer simulation, the tool-chip thermal contact is perfect with heat transfer coefficient (*h_c_*)
- The boundaries of the work piece and tool are set at room temperature (*T*_∞_ = 25 °C) far from the cutting zone
- The convection heat transfer coefficient is *h*_∞_ = 20 W/m^2^ °C

The value of the convection heat transfer coefficient *h_c_* considered for air flow was based on the velocity of the surrounding air and approximated according to the following equation:

*h*_*c*_ = 10.45 − *v* + 10 *v*^1/2^ (5)

where *v* is the relative velocity between object surface and air (m/s).

It should be noted that during the cutting process, heat is generated in the cutting zone. At the same time, natural cooling occurs, because of a change in temperature due to the ambient air. The cooling mechanism can be expressed according to Newton’s law of cooling as follows:

(6) $$k_{i}\Delta T=h\left(T_{air}-T\right)$$

where *k_i_*, Δ*T*, *h* and *T_air_* represent, respectively, the thermal conductivity of the considered material, the temperature gradient, the heat transfer coefficient for natural convection, and the ambient temperature.

### 2.2. Numerical Design Planning

This section is dedicated to the numerical method that was adopted in the present study. Orthogonal cutting tests were performed to study the interactive effects of different cutting nose geometries on milling process performance under different cutting conditions.

#### Design of Experiments

In order to study the interactive effects of different machining parameters with tool nose geometries on machining process performance, it is necessary to consider the importance of cutting conditions in identifying the main effects and the interactions of parameters. Only round tool geometry was considered. Table 3 presents the design of experiments used in the numerical simulations. The clearance angle was constant at α = 15°.

For the first set of experiments, only the nose radius and the cutting speed were varied; in the second set, the feed and the nose radius were varied; for the third set, the rake angle of the tool and the nose radius were varied, and, finally, for the fourth set of tests, only the nose radius was varied.

## 3. Numerical Simulation Results

**Figure 4:**
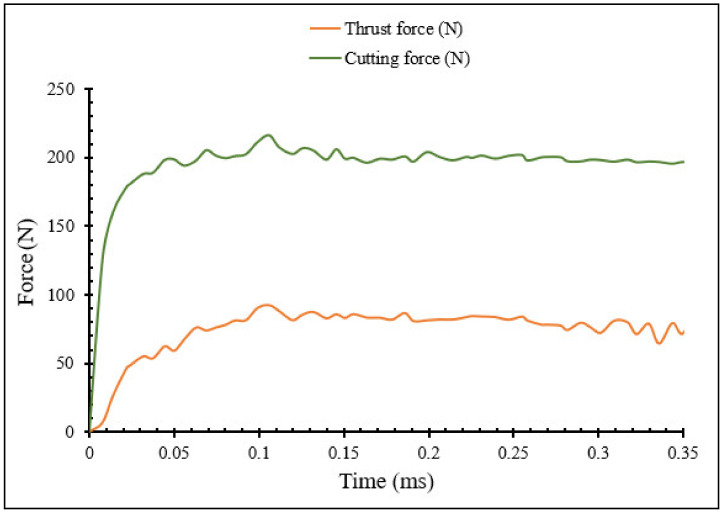
Transient force simulations during machining process at *V* = 400 m/min, *r_c_* = 0.3 mm, *f* = 0.1 mm/rev, *γ* = 5°.

**Figure 5:**
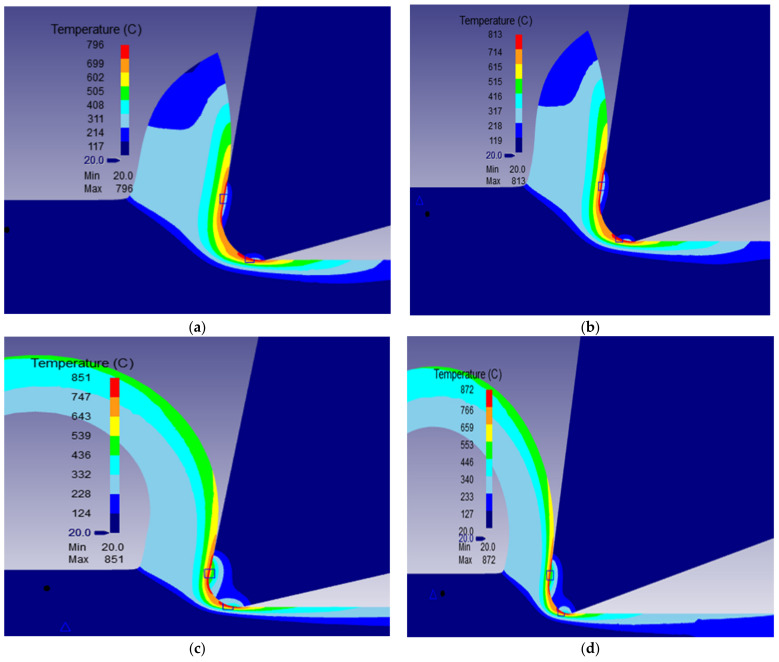
Distribution of cutting temperature in the workpiece and cutting tool at *V* = 400 m/min, *f* = 0.1 mm/rev, *γ* = 5° for the nose radii *r_c_*: (**a**) 0.3 mm, (**b**) 0.5 mm (**c**) 0.7 mm and (**d**) 0.9 mm.

**Figure 6:**
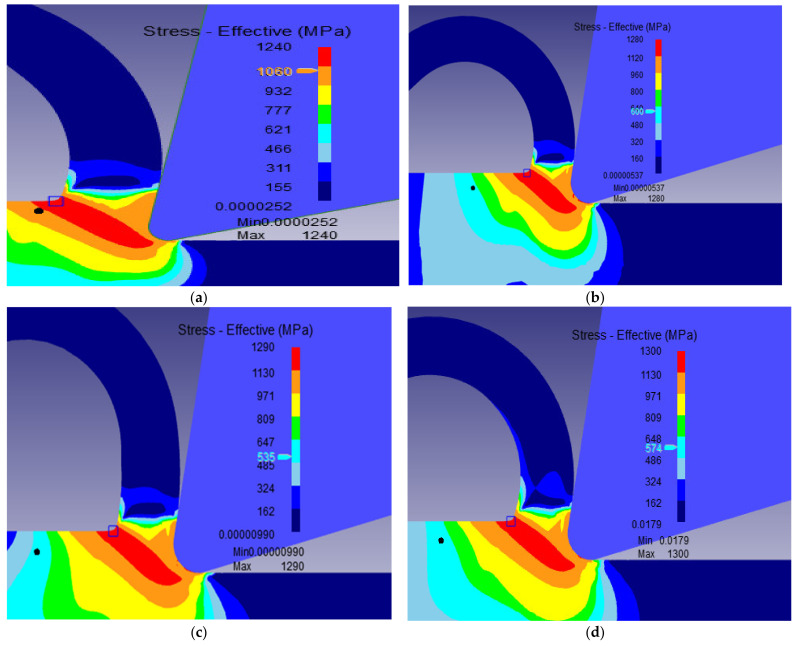
Distribution of effective stress in the workpiece and cutting tool at *V* = 400 m/min *f* = 0.1 mm/rev, *γ* = 5° for the nose radii *r_c_*: (**a**) 0.3 mm, (**b**) 0.5 mm (**c**) 0.7 mm and (**d**) 0.9 mm.

**Figure 7:**
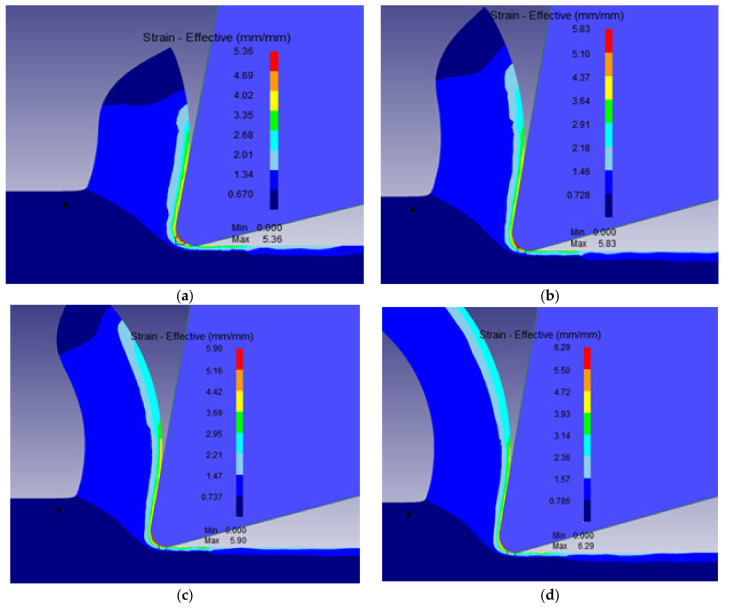
Distribution of effective strain in the workpiece and cutting tool at *V* = 400 m/min *f* = 0.1 mm/rev, *γ* = 5° for the nose radii *r_c_*: (**a**) 0.3 mm, (**b**) 0. 5 mm (**c**) 0.7 mm and (**d**) 0.9 mm.

The different machining properties were simulated numerically using the transient analysis module of DEFORM 2D software based on the two-dimensional (2D) Finite Element Method (FEM). For cutting forces, the transient proposed model enables determination of the evolution of the machining forces with time. Figure 4 illustrates the variation of machining force, namely the cutting force and thrust force, during the machining process for *V* = 400 m/min, *r_c_* = 0.3 mm, *f* = 0.1 mm/rev, *γ* = 5°. Figure 5, Figure 6 and Figure 7 show the numerical simulation results of cutting temperature, effective stress and effective strain for nose radii (0.3, 0.5, 0.7 and 0.9 mm) at t = 0.0014 s in the workpiece, cutting tool, and at the interface. For cutting temperatures, the simulation results illustrate the distribution of temperatures in different locations in the model.

From Figure 4, the cutting forces and thrust forces are extracted in the X and Y directions, respectively. For each nose radius, the numerical machining forces were obtained from the same simulation time until the steady state, when the transient variations of the cutting force are higher than those of the thrust forces.

From the distribution of cutting temperature displayed in Figure 5, it can be seen that the cutting temperatures were higher in the contacts areas (between the tool and in the chips, between the tool and the workpiece). Also, the higher the tool nose radius was high the higher was the maximum cutting temperature. This is a consequence of higher stresses (Figure 6) and strains (Figure 7) taking place in the workpiece in the shearing zones and frictions at tool-chip and tool-workpiece interfaces.

Figure 6 presents the simulation of the distribution of the stresses in the workpiece and tool. The results in Figure 6 reveal that cutting stresses were developed in the machining direction due to forces applied in the X direction (Figure 4). As expected, maximum stresses occurred in the shear plane. The higher the tool nose radius was the more maximum stress was exerted in the workpiece and the higher the shear plane. It reached 1300 MPa for a nose radius of 0.9 mm as compared to 1280 MPa for a nose radius of 0.3 mm. These high stresses, and the subsequent deformations, were responsible for the high temperatures already observed in the cutting zones.

Figure 7a–d present the effective strain profiles predicted in the cutting zone for the given tool nose radii. Maximum strain occurred at the tool tip followed by the secondary deformation zone (tool-chip contact zone). As expected, these strains increased slightly with increase in the tool nose radius. For an increase of tool nose radius from 0.3, to 0.5, 0.7 and 0.9 mm, there were increases in the effective strains of 9%, 10% and 17%, respectively. This is a consequence of change in flow stress and in cutting stresses when tools with large nose radii are used.

**Figure 8:**
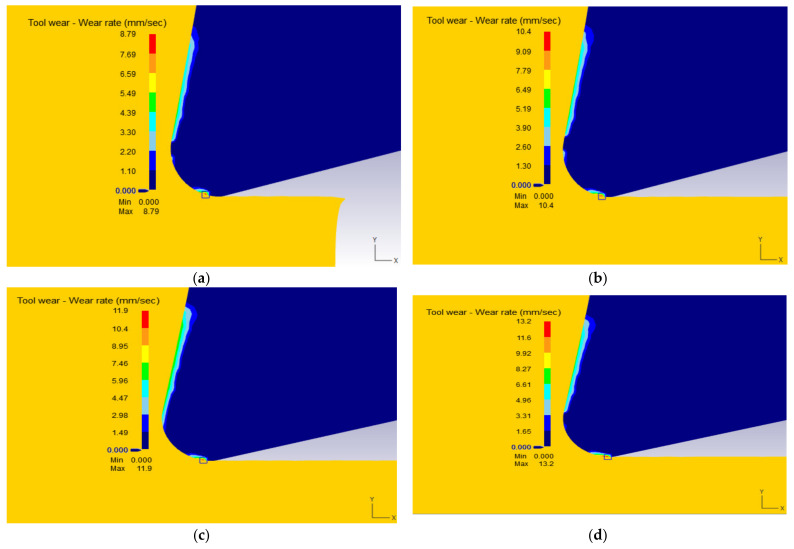
The simulation results for tool wear rate at *V* = 400 m/min, *f* = 0.1 mm/rev, *γ* = 5° for the nose radii *r_c_*: (**a**) 0.3 mm, (**b**) 0.5 mm (**c**) 0.7 mm and (**d**) 0.9 mm.

Figure 8 displays the simulated tool wear rate distribution as a function of tool nose radii. The wear rates are higher in the rake face of the tool (middle of the tool-chip contact length) and at the tool tip interface where the effective rake angle is smallest. In fact, the more the effective tool rake angle is negative, the higher the temperature (Figure 5), the flow stress and strain. Consequently, the tool wears out quicker. Wear increased with tool nose radii. For example, for an increase of tool nose radius from 0.3, to 0.5, 0.7 and 0.9 mm, there were increases on effective strains of 14%, 35% and 50%, respectively.

**Figure 9:**
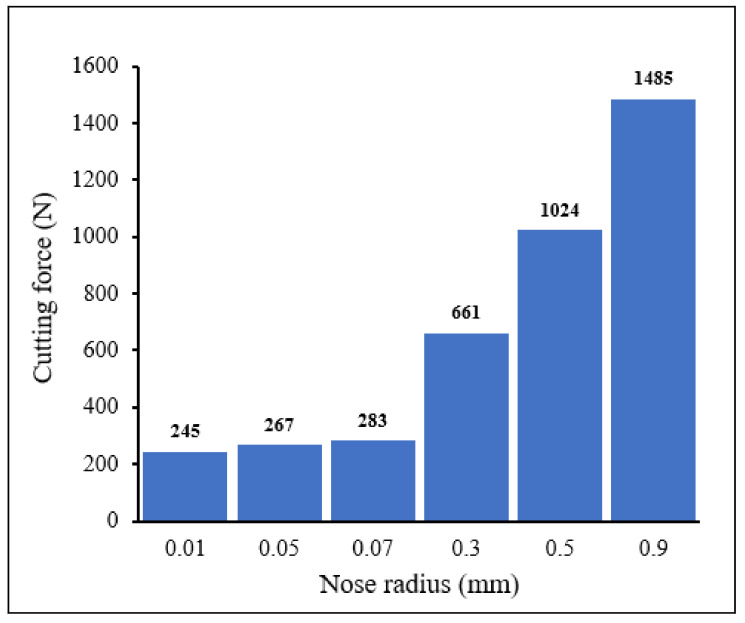
Distribution of cutting force for different tool nose radii *V* = 104 m/min, *f* = 0.1 mm/rev, *γ* = 15°.

**Figure 10:**
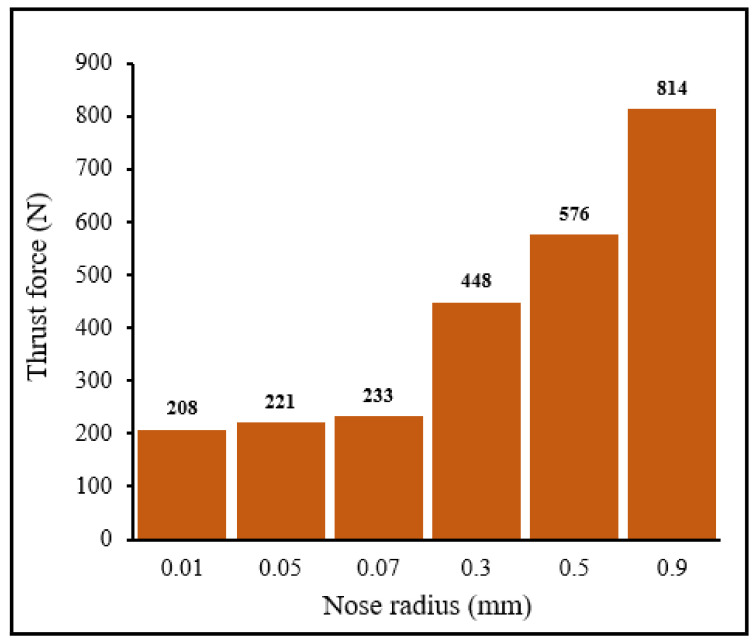
Distribution of thrust force for different tool nose radii *V* = 104 m/min, *f* = 0.1 mm/rev, *γ* = 15°.

Figure 9 and Figure 10 display the evolution of cutting forces (Figure 9) and thrust force (Figure 10) when machining with tools with different nose radii. These graphs were extracted from figures, such as the one presented in Figure 4. As expected, cutting forces were higher than thrust forces. In general, the forces were low for small tool nose radii (0.01–0.07 mm) and high when large-nose radii were present (0.3–0.9 mm). These results are in good agreement with literature (See comparisons in Table 4 and Table 5). When the tool is almost sharp (very small nose radii), the resistance to the shearing action is limited and less power and energy are required for the cutting process. On the contrary, when large nose radii are used, resistance to cutting action is high and so is the cutting force, and thus the power requirement. It should be noted that tools with high nose radii generate a better part surface finish and compressive residual stresses that could be beneficial for field performance of the machined part.

From Figure 9 and Figure 10, it may be noted that both the cutting force and thrust force increased slightly as the nose radius increased. By increasing the edge radius 7 times (from 0.01 to 0.07 mm), the cutting force and the thrust force increased by about 15.5% and 12%, respectively. This is due to cutting nose geometry, which requires greater forces for shearing the material. As the nose radius increased, the chip thickness increased and the shear angle decreased, which led to the development of a large shear plane in the deformation area, thereby increasing cutting forces. Further increase in the nose radius from 0.3 to 0.9 mm, resulted in significant increase in the thrust and cutting forces, of about 82% and 125%, respectively.

The cutting forces are higher than the thrust forces, due to the fact that the cutting force is applied in the direction of primary motion. Afrasiabi et al. (2021) have found similar results on a numerical-experimental study of orthogonal cutting of AISI 1045 machined at cutting speeds ranging from 60 m/min to 180 m/min [14]. It is known that the cutting force comprises a large part of the total force and contributes in determining the global power necessary for the machining process.

**Figure 11:**
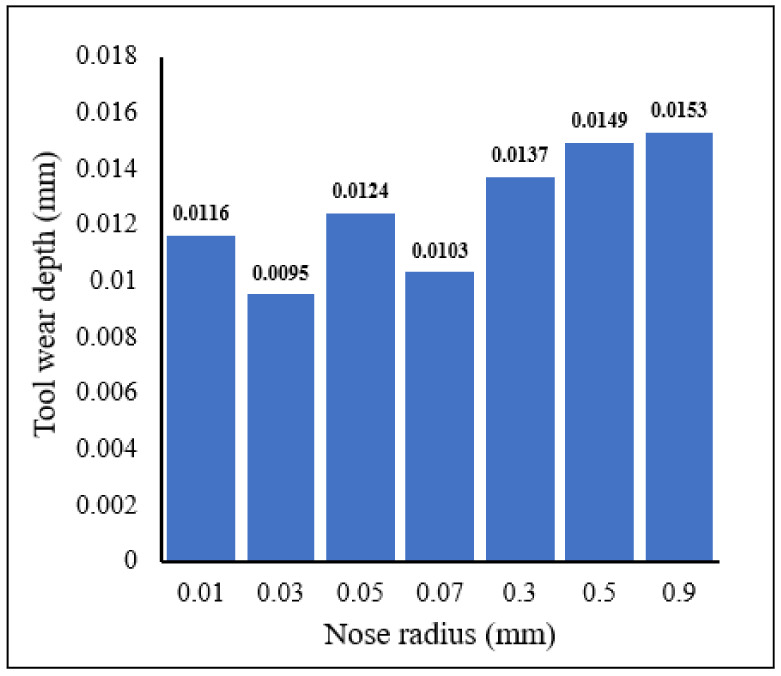
Distribution of tool wear for different tool nose radii *V* = 100 m/min, *f* = 0.1 mm/rev, *γ* = 5°.

Figure 11 shows the distribution of tool wear for different tool nose radii for *V* = 100 m/min, *f* = 0.1 mm/rev, *γ* = 5°. It can be noticed from Figure 11 that tool wear depth in the rake face of the tool does not directly depend on variation of nose radius, especially for the small tool nose radius scale. The wear width had approximately the same values for 0.03 mm and 0.07 mm radii. For 0.01 mm and 0.05 mm radii, the difference was about 7%. For large nose radius scale the tool wear width increased with increase in the nose radius. The wear widths for the large nose radius scale were higher than those obtained for the small nose radius scale. An indirect interaction between the cutting speed, or the tool rake angle, and the tool nose radius are expected on the tool wear rake as these interactions affect the cutting stress and/or the cutting temperature; these aspects are analysed later in the current article (Section 3.2 Interactive effects between cutting parameters and tool geometries).

### 3.1. Comparison of Results between Numerical Simulations and Experimental Data from Literature

In order to validate the numerical simulation tests, the obtained results were compared with experimental data [5,15]. Experimental research mentioned in [5] investigated the effects of tool nose preparation on cutting forces, power, and tool wear in machining of AISI 1045 steel prepared with only honed nose geometry (*r_c_* = 0.03 mm), by adopting the method of Taguchi. The second experimental research [15] covered a study of the effects of tool edge on cutting forces, temperatures, stresses and tool wear in machining of AISI 1045 steel prepared with three honed edge geometries (0.25, 0.5, 0.75 mm). Later on, other numerical simulation tests were realised for large tool nose radii (0.2, 0.5, 0.7 mm) by considering the same machining conditions. Table 4 and Table 5 present the obtained numerical results compared to experimental results for small and large tool nose radii, respectively.

**Table 4:** Comparison of numerical and experimental results for small tool nose radius.

	Numerical Results	Experimental Data [5]	Error
	Nose Radius (mm): 0.03		
Cutting temperature (°C)	507	465	8%
Cutting stress (MPa)	1271	1318	4%
Cutting forces (N)	258	243	6%

**Table 5:** Numerical and experimental results comparison for large tool nose radius.

	Numerical Results			Experimental Data [15]			Error (*r_c_* = 0.5) %
	Nose Radius (mm)						
	0.2	0.5	0.7	0.25	0.5	0.75	
Cutting temperature (°C)	716	814	844	657	682	627	16.2%
Cutting stress (MPa)	1239	1241	1237	1230	1200	1200	3.3%
Cutting forces (N)	367	562	604	519.4	481	462	14.4%
Tool wear width (mm)	0.012	0.0142	0.015	0.013	0.0147	0.010	3.5%

The computed errors between the numerical and experimental results for the small tool nose radius varied from 4% to 8% (See Table 4). For large tool nose radii, the calculated errors varied from 3% to 16% only for *r_c_* = 0.5 (see Table 5). These errors could be due to the combination of mesh, the model, and convergence of the process simulation. The error for cutting temperature was due to loss of heat by convection which could not be considered during the machining process.

### 3.2. Interactive Effects between the Cutting Parameters and the Tool Geometries

#### 3.2.1. Influence of Feed Rate and Tool Nose Radius on Cutting Temperature and Cutting Stresses

**Figure 12:**
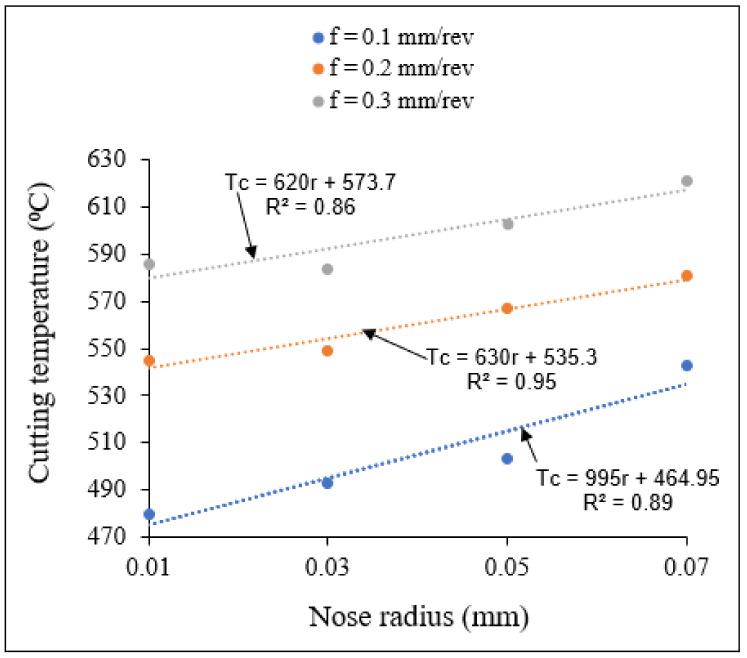
Variation of cutting temperature with small tool nose radius for different feed rates at *V* = 400 m/min, *γ* = 5°.

**Figure 13:**
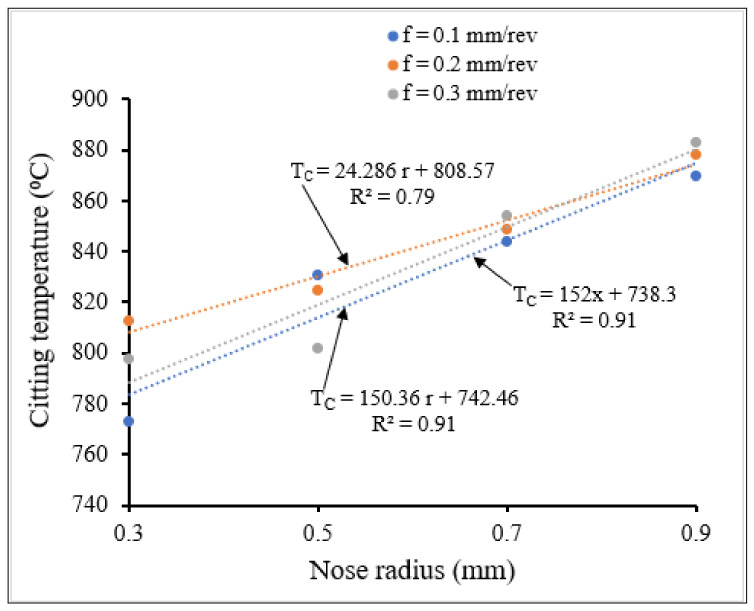
Variation of cutting temperature with large tool nose radius for different feed rates at *V* = 400 m/min, *γ* = 5°.

Figure 12 and Figure 13 show the variation of maximum temperature with small and large nose radii, respectively, for different feed rates. The obtained cutting temperatures and stresses can be approximated by linear equations, which can be established using appropriate statistical models (linear regression models).

From Figure 12 and Figure 13 the cutting temperatures were observed to increase with increasing feed rate, due to increase in frictional heat resulting from an increasing tool-chip contact zone with increasing feed rate. Similarly, the cutting temperatures increased when tool nose radius increased, for all the feed rates studied. While the temperatures for the large nose radii were higher than those noted for the small nose radius range, the slopes of the temperature-nose radius lines are lower for the large nose radius scale in comparison.

**Figure 14:**
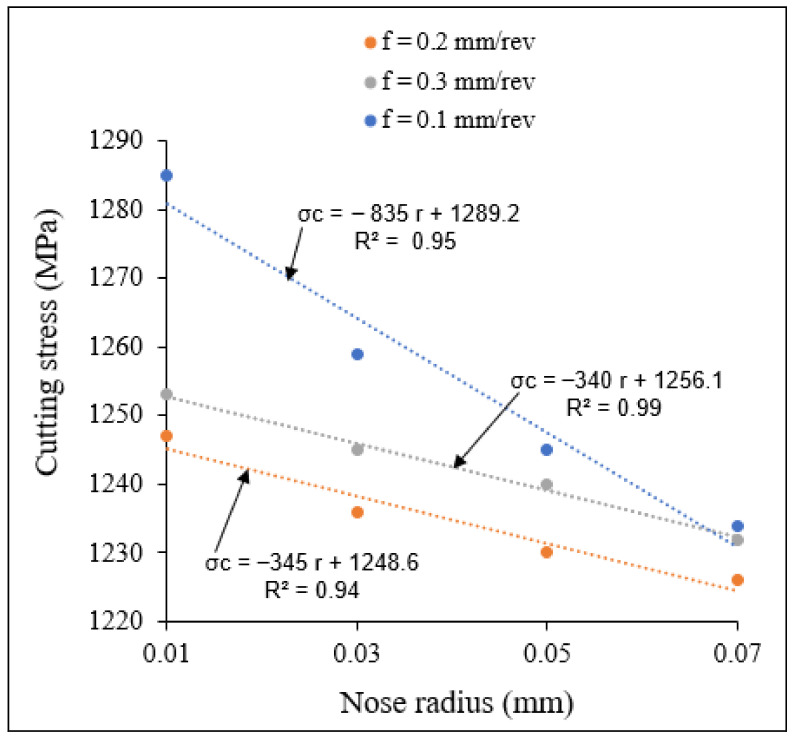
Variation of cutting stress with small tool nose radius at different feed rates at *V* = 104 m/min, *γ* = 5.

**Figure 15:**
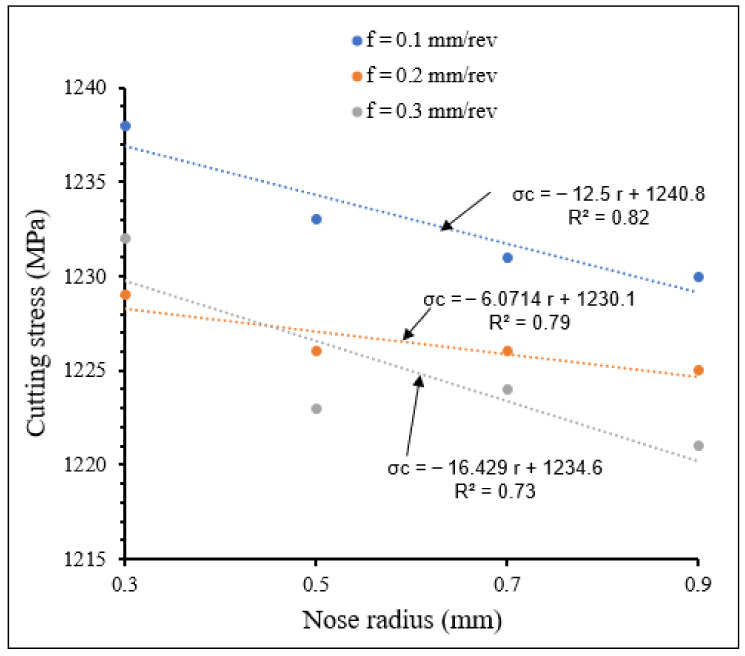
Variation of cutting stress with large tool nose radius at different feed rates. *V* = 104 m/min, *γ* = 5.

Figure 14 and Figure 15 show the variation in cutting stress with small and large nose radii, respectively, at different feed rates. From these figures, it is observed that for both small and large tool nose radius scales, the cutting stresses decreased with increasing feed rate for all nose radii studied, and also decreased with tool nose radius for all the considered feed rates. The slopes of cutting stress for the small nose radius scale are higher than those for the large nose radius scale.

#### 3.2.2. Influence of Cutting Speed and Tool Nose Radius on Cutting Temperature and Cutting Stresses

**Figure 16:**
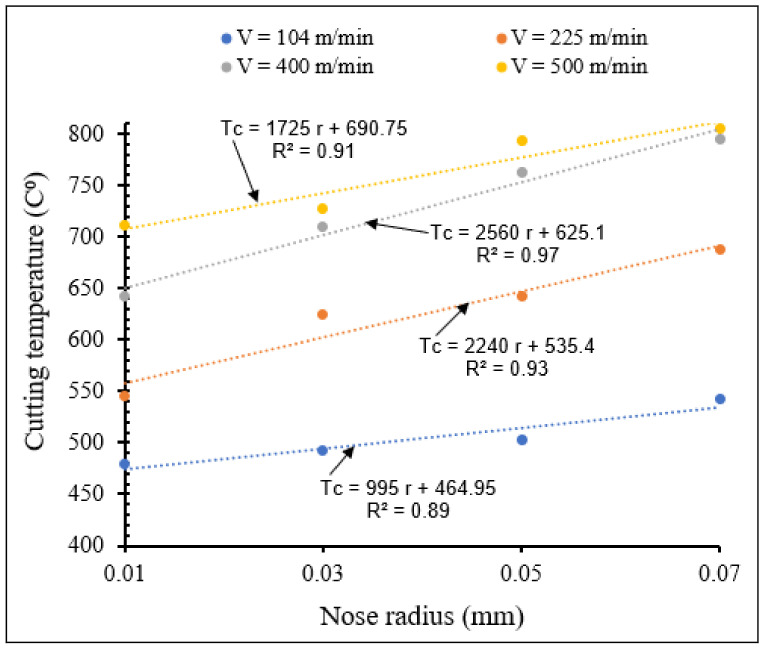
Variation of maximum temperature with small tool nose radius for different cutting speeds at *f* = 0.1 mm/rev, *γ* = 5°.

**Figure 17:**
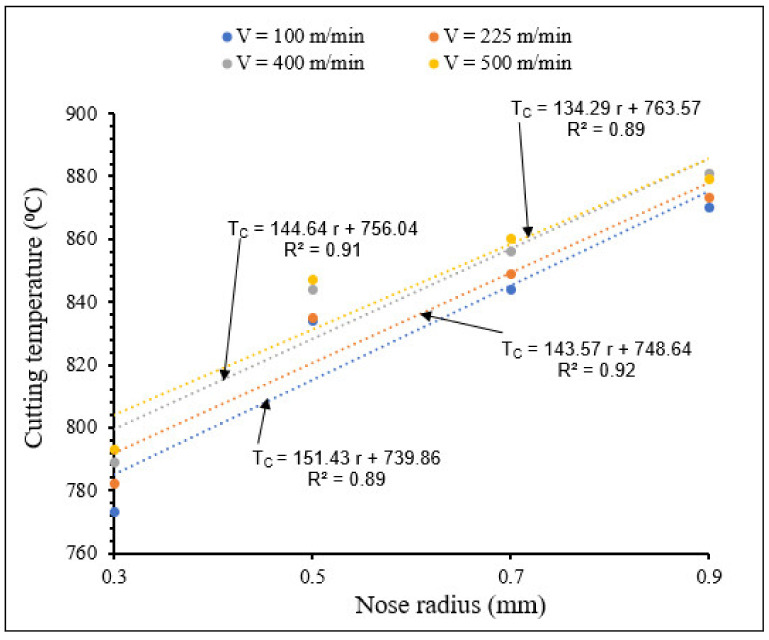
Variation of maximum temperature with large nose radius for different cutting speeds at *f* = 0.1 mm/rev, *γ* = 5°.

Figure 16 and Figure 17 show the effects of the combination of cutting speed and tool nose radius on cutting temperature for tools with small- and large-scale nose radii, respectively.

From Figure 16 and Figure 17 it can be seen that for all the applied cutting speeds, the cutting temperature increased when nose radius increased (small and large scale), due to large friction and plastic heat, as explained before. For the same nose radius, cutting temperature increased when cutting speed increased. There was a significant increase of cutting temperature with large nose radii tools, compared to that observed when using cutting tools with small nose radii.

**Figure 18:**
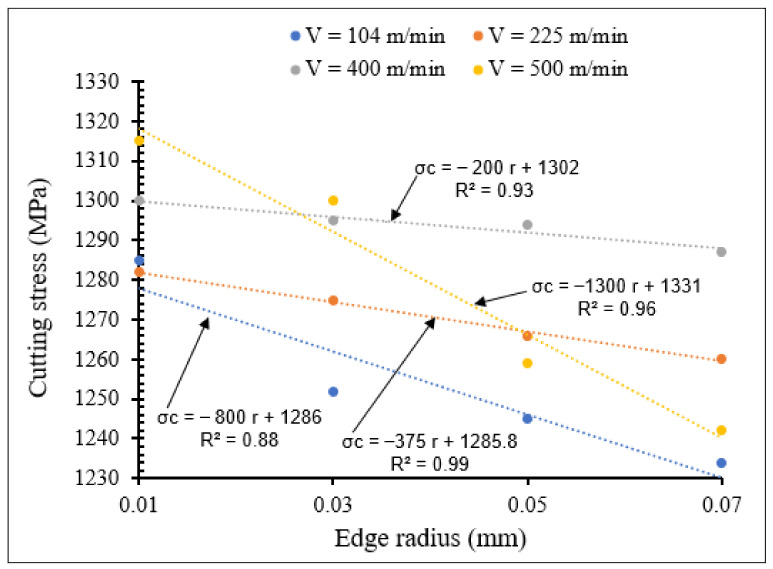
Variation of cutting stress with small tool nose radius for different cutting speeds at *f* = 0.1 mm/rev, *γ* = 5°.

**Figure 19:**
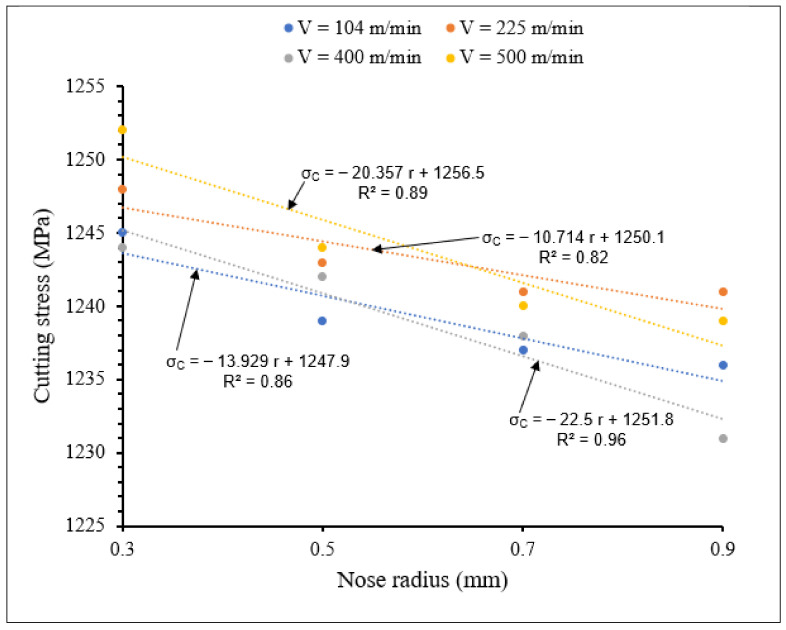
Variation of cutting stress with large tool nose radius for different cutting speeds at *f* = 0.1 mm/rev, *γ* = 5°.

Figure 18 and Figure 19 display the variation in cutting stress for different cutting speeds, using tools with small and large nose radii, respectively.

From Figure 18 and Figure 19 it is noted that at all the applied cutting speeds, the cutting stresses decreased with increase in the nose radius, for both small and large nose radii scales. The sharpest decrease in cutting stress was observed at the cutting speed of 500 m/min.

All these obtained results can be validated by previous data in literature during cutting comparable AISI 1045 steel materials, showing the influence of tool edge radius on machining temperature [16,17].

For all the nose radii the cutting stress decreased with increasing cutting speed. These decreases can be observed by the sign (−) in the established linear equations. For small tool nose radius and for higher cutting speed, especially *V*_c_ = 500 m/min, the slope of the curve is much larger due to the larger frictional and plastic heat generated during the cutting process, which results in less cutting forces and then in smaller cutting stresses. The cutting stresses for the small scale are higher than those for the large scale. Therefore, the more the nose tool radius increased, the more the cutting stress variations increased. The same results were found by Shravankumar C. and Bharat. S. Kodli [18], who demonstrated that tool radius has inversely proportional effects on cutting stress.

#### 3.2.3. Influence of Rake Angle and Tool Nose Radius on Cutting Temperature and Cutting Stresses

**Figure 20:**
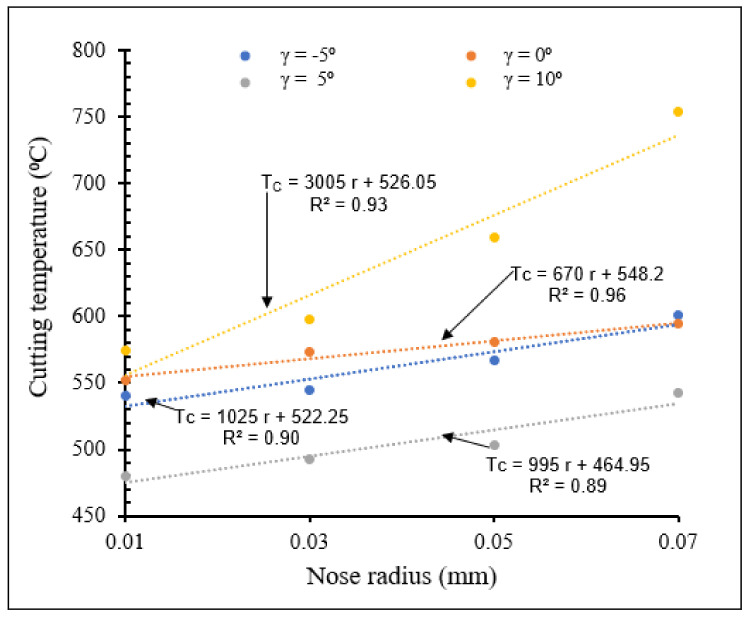
Variation of maximum temperature with small tool nose radius for different rake angles at *V* = 104 m/min, *f* = 0.1 mm/rev.

**Figure 21:**
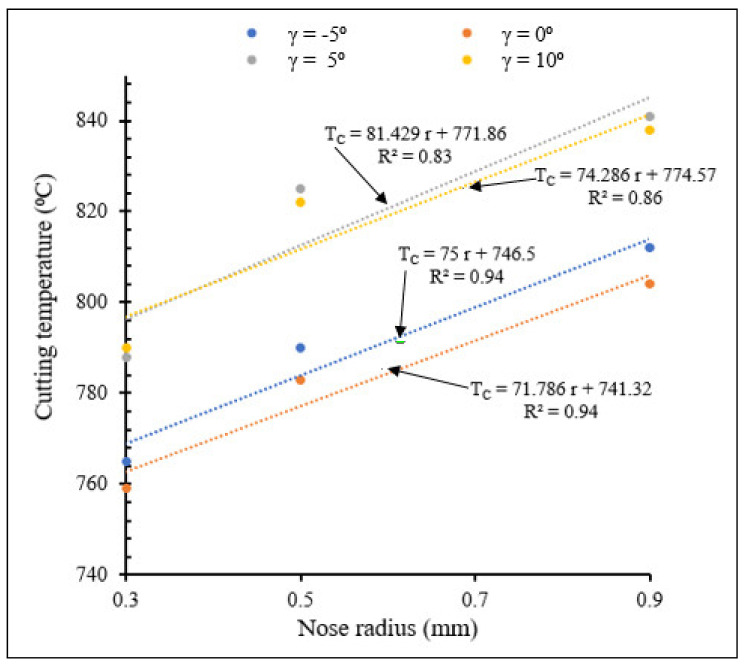
Variation of cutting temperature with large tool nose radius for different rake angles at *V* = 400 m/min, *f* = 0.1 mm/rev.

Figure 20 and Figure 21 show the effects of the combination of rake angle and tool nose radius on cutting temperatures for small- and large-scale tool nose radii, respectively.

From Figure 20 and Figure 21, for both small and large nose radii, cutting temperatures increased with increasing rake angle except for the values of 5° for small scale and 0° for large scale, due to increase of the contact force between chip and cutting tool. For all rake angles, cutting temperatures increased when tool nose radius increased. The more the nose radius increased, the more the heat volume generated in the contact area increased. The cutting temperatures for large tool nose radii were higher than those observed with small tool nose radii. However, the slopes of the Tc-r lines indicate that the cutting temperatures rise faster with increasing nose radius in the latter case than when tool nose radii are large.

**Figure 22:**
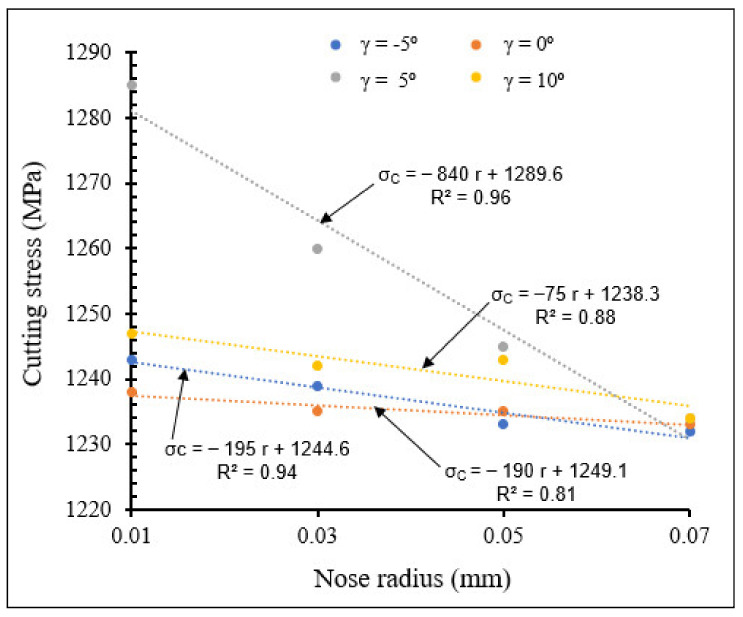
Variation of cutting stress with small tool nose radius for different rake angles at *V* = 104 m/min, *f* = 0.1 mm/rev.

**Figure 23:**
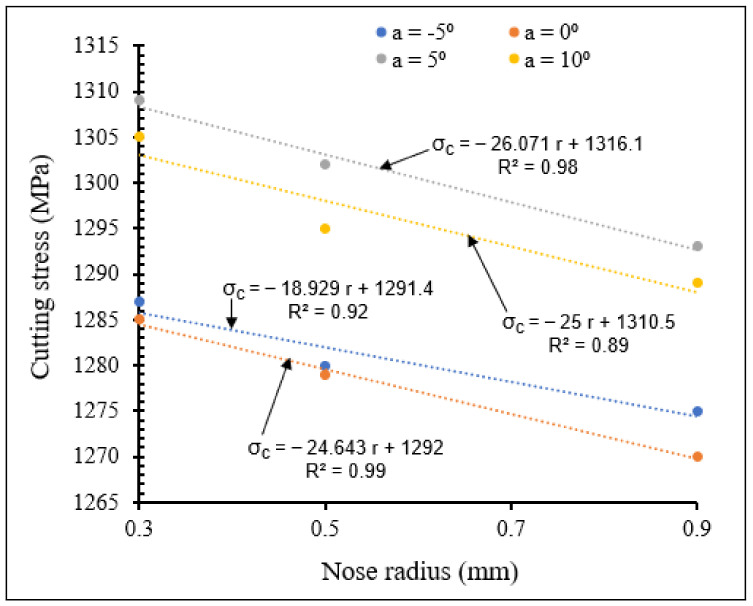
Variation of cutting stress with large tool nose radius for different rake angles at *V* = 104 m/min, *f* = 0.1 mm/rev.

Figure 22 and Figure 23 show the influence of the combination of rake angle and tool nose radius on cutting stress for small and large tool nose radius scales, respectively.

Taking all the tool nose radius values investigated into account, Figure 22 and Figure 23 reveal that when rake angle increased, cutting stress decreased for both small and large tool nose radius scales, due to reduction in chip-tool contact pressure, causing decrease of cutting forces and therefore reduction in cutting stress. For all the applied rake angles, the cutting stress decreased when nose radius increased. The cutting stresses were higher for large scale than for small scale. However, large slopes are observed for small scale, especially for a rake angle of 5°.

#### 3.2.4. Influence of Cutting Speed and Nose Edge Radius on Tool Wear

**Figure 24:**
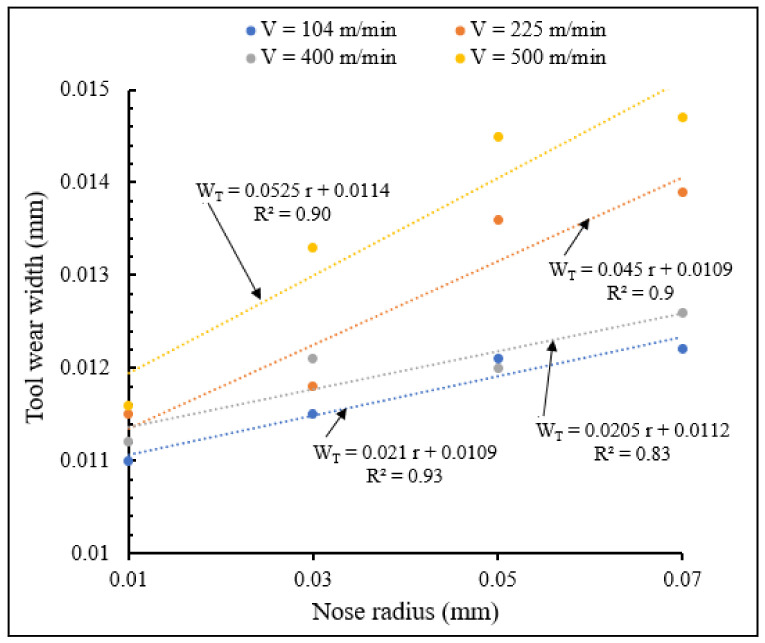
Variation of tool wear width with small nose radius for different cutting speeds at *f* = 0.1 mm/rev, *γ* = 5°.

**Figure 25:**
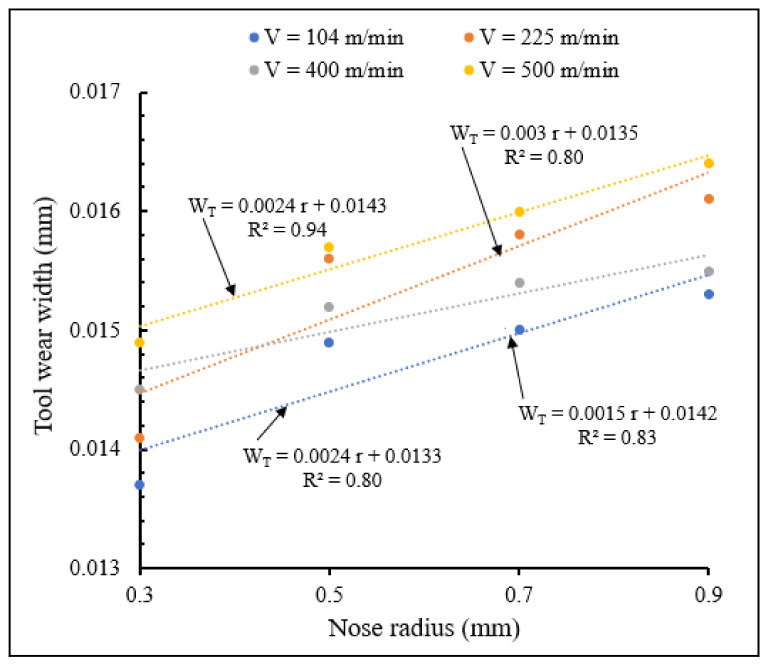
Variation of tool wear width with large nose radius for different cutting speeds at *f* = 0.1 mm/rev, γ = 5°.

Figure 24 and Figure 25 illustrate the variations in tool wear width with nose radius for different cutting speeds, for small and large tool nose radius scales, respectively showing the interactive influence of cutting speed and tool nose radius on tool wear. Strong interaction was obtained for small nose scale and for a cutting speed of 500 m/min where there is the sharpest slope of the linear equation of tool wear approximated by trend.

It is observed from these figures that wear width increased when nose radius increased for different cutting speeds and for both small and large nose radii. Similar results were obtained in previous works conducted by T. M. El-Hossainy et al., Xingzhong, Z. et al. [19,20], which revealed that increasing cutting speed results in increasing temperature, leading to an increase in tool wear. For the same nose radius, wear width increases when cutting speed increases. Wear width variations for large nose radius are more important than those of small radius scale. The increase of nose radius and cutting speed increase tool wear

#### 3.2.5. Influence of Rake Angle and Tool Nose Radius on Tool Wear

**Figure 26:**
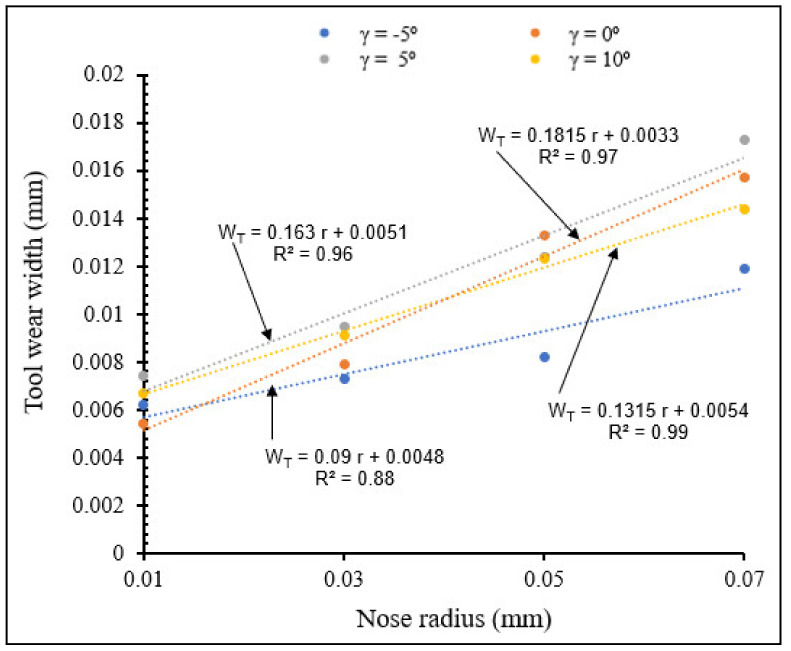
Variation of tool wear width with nose radius for different rake angles at *V* = 104 m/min, *f* = 0.1 mm/rev.

**Figure 27:**
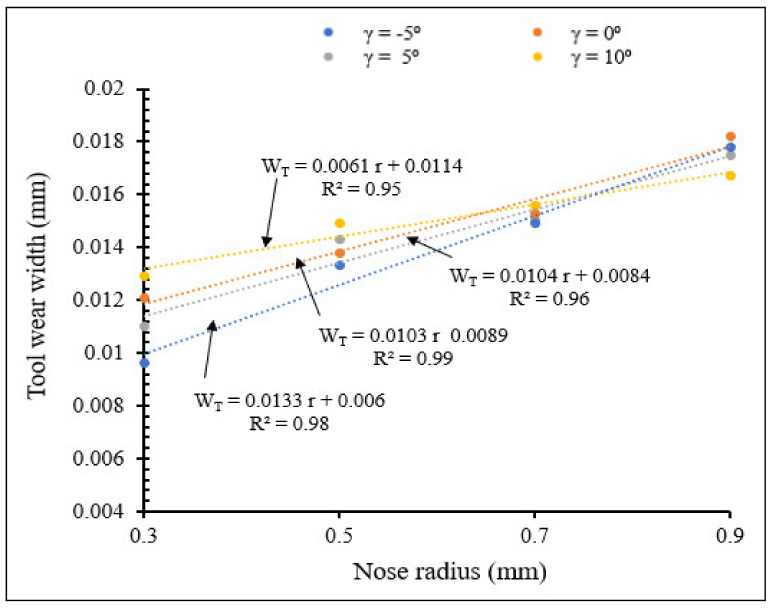
Variation of tool wear width with large tool nose radius for different rake angles at *V* = 104 m/min, *f* = 0.1 mm/rev.

Figure 26 and Figure 27 illustrate the variation of tool wear width with nose radius for different rake angles, for small and large nose radius ranges, respectively, showing the interactive influence between rake angle and tool nose radius on flank wear.

From Figure 26 and Figure 27 tool wear width increased when nose radius increased for different rake angles, for both small and large nose radii. For the same small nose radius, wear width increased when rake angle increased from −5° to 5°, but it decreased from rake angle 5° to 10°. For the same large nose radius, wear width increased when rake angle increased. This is due to accumulation of chips near the tool surface, causing increase of machining force, heat generated and consequent increase in tool wear. These results can also be validated by T. M. El-Hossainy et al., Xingzhong, Z. et al. [19,20]. The tool wear width comparison between small and large nose radius showed that there is no significant difference in the results variation.

## 4. Conclusions

A numerical simulation model, based on the finite element method, was developed for predicting the effects of tool nose radius on cutting temperature, machining stress and tool wear in the orthogonal milling of AISI 1045 steel, using various cutting process parameters. The model was validated using data from literature. From an analysis of the results obtained, the following conclusions were drawn.

- In general, for all the tested cutting conditions and for the studied range of nose radii (from 0.01 to 0.07 mm, and from 0.3 to 0.9 mm) cutting temperature, stress and tool wear vary approximately linearly with tool nose radius.
- Tool nose radius was found to influence cutting forces, cutting stress, strain, temperature in the cutting zones and, finally, tool degradation. Tool degradation (wear rate) was high on the tool rake face and at the tool tip in the region where the effective rake angle was the smallest.
- Interactions between tool nose radius and cutting parameters (speed, feed) affected mostly cutting stress and, slightly, tool wear rate. These interactions did not much influence cutting temperature, that was found to be high when the tool nose radius and/or the cutting speed were high.
- Maximum temperature was found to take place at the middle of tool-chip contact length and at the interaction of nose radius and flank face of the tool. Except for some fluctuations, there was no significant difference in tool wear rate between small and large nose radius scales.
- The results obtained from this study and their analysis will permit future research to determine the optimal geometrical parameters for improving machining process characteristics, using appropriate optimisation methods, and to establish cutting empirical formulae that can be useful for machining industrial applications.

## Figures and Tables

**Table 1:** Thermal and physical properties of workpiece and tool materials (adapted from [11,12]).

Item	Tool	Workpiece
Properties	Uncoated cemented carbide	AISI 1045
Density (kg/m^3^)	11,900	7870
Thermal conductivity (W/m °C)	50	45
Specific heat (J/kg °C)	375	590

**Table 2:** Mechanical properties of workpiece and tool materials [12].

Item	Tool	Workpiece
Properties	Uncoated cemented carbide	AISI 1045
Young’s modulus (GPa)	620	200
Poisson ratio	0.26	0.29
Hardness (HB)	93	163

**Table 3:** Design for numerical simulations.

	Tool Geometry and Machining Parameters			
Simulation Group ID	Nose Radius *r_c_* (mm)	Rake Angle *γ* (°)	Cutting Speed *V* (m/min)	Feed Rate *f* (mm/rev)
1	0.01–0.9	5	100, 104, 225, 250, 400, 500	0.1
2			100	0.1, 0.2, 0.3
3		−5, 0, 5, 10		0.1
4		5		0.1

## Data Availability

Data available on request due to restrictions privacy or ethical.

## Nomenclature

**Symbol**	**Unit**	**Description**
*r_c_*	mm	Nose radius
α	deg	Clearance angle
*γ*	deg	Rake angle
S_α_	Cutting edge segment on the flank face	
S_β_	Profile flattening	
S_γ_	Cutting edge segment on the rake face	
σ_c_	Cutting stress	
